# How to study improvement interventions: a brief overview of possible study types

**DOI:** 10.1136/bmjqs-2014-003620

**Published:** 2015-03-25

**Authors:** Margareth Crisóstomo Portela, Peter J Pronovost, Thomas Woodcock, Pam Carter, Mary Dixon-Woods

**Affiliations:** 1 Social Science Applied to Healthcare Research (SAPPHIRE) Group, Department of Health Sciences, School of Medicine, University of Leicester, Leicester, UK; 2 Department of Health Administration and Planning, National School of Public Health, Oswaldo Cruz Foundation, Rio de Janeiro, RJ, Brazil; 3 Departments of Anesthesiology, Critical Care Medicine, and Surgery, Armstrong Institute for Patient Safety and Quality, School of Medicine, and Bloomberg School of Public Health, Johns Hopkins University, Baltimore, Maryland, USA; 4 NIHR CLAHRC for Northwest London, Imperial College London, Chelsea and Westminster Hospital, London, UK

**Keywords:** Statistical process control, Social sciences, Quality improvement methodologies, Health services research, Evaluation methodology

## Abstract

Improvement (defined broadly as purposive efforts to secure positive change) has become an increasingly important activity and field of inquiry within healthcare. This article offers an overview of possible methods for the study of improvement interventions. The choice of available designs is wide, but debates continue about how far improvement efforts can be simultaneously practical (aimed at producing change) and scientific (aimed at producing new knowledge), and whether the distinction between the practical and the scientific is a real and useful one. Quality improvement projects tend to be applied and, in some senses, self-evaluating. They are not necessarily directed at generating new knowledge, but reports of such projects if well conducted and cautious in their inferences may be of considerable value. They can be distinguished heuristically from research studies, which are motivated by and set out explicitly to test a hypothesis, or otherwise generate new knowledge, and from formal evaluations of improvement projects. We discuss variants of trial designs, quasi-experimental designs, systematic reviews, programme evaluations, process evaluations, qualitative studies, and economic evaluations. We note that designs that are better suited to the evaluation of clearly defined and static interventions may be adopted without giving sufficient attention to the challenges associated with the dynamic nature of improvement interventions and their interactions with contextual factors. Reconciling pragmatism and research rigour is highly desirable in the study of improvement. Trade-offs need to be made wisely, taking into account the objectives involved and inferences to be made.

## Introduction

Improvement interventions, which can be defined broadly as purposeful efforts to secure positive change, have become an increasingly important focus of activity within healthcare.^1^ How improvement interventions can best be studied, however, has remained contested; as with most new fields, many of the key terms, concepts and techniques currently escape consensus. In a rapidly evolving field, and with the task of designing, testing, implementing and evaluating quality improvement interventions, as well as producing generalisable knowledge growing in complexity,^2^ it is helpful to characterise the kinds of study designs that can be used to study improvement interventions. This is the task to which this paper is directed; it is intended to offer an introductory overview and bibliography, particularly for those new to the field. It is based on a narrative literature review^3^ using English language articles selected through a systematic search strategy (box 1) and reflection based on our experience in the field.

Box 1 **Literature search strategies employed**

**Search in institutional sites:**

- The Health Foundation (http://www.health.org.uk)
- Institute of Healthcare Improvement (http://www.ihi.org)
- Improvement Science Research Network (http://www.isrn.net)

**Bibliographic search in PUBMED - articles published in English from 2005:**

Based on terms:

‘improvement science’; ‘implementation science’; ‘translational research’; ‘science of quality improvement’; ‘quality improvement research’; ‘improvement science and context’; ‘improvement science and theories’; ‘healthcare quality improvement interventions’; ‘designing and evaluating complex interventions’; ‘quality improvement evaluation’; ‘improvement science methods’; ‘implementation science methods’; ‘healthcare quality improvement intervention clinical trials’; ‘healthcare quality improvement intervention effectiveness’; ‘healthcare quality improvement intervention observational studies’; ‘healthcare quality improvement intervention economic evaluations’; ‘healthcare quality improvement intervention cost-effectiveness’; ‘healthcare quality improvement intervention literature reviews’; ‘healthcare quality improvement intervention sustainability’.

Based on authors with extensive production in the field

**References identified in the papers selected based on the other strategies, independently of their date.**

## Studying improvement in healthcare

We begin by noting that a significant body of work in the area of improvement has taken the form of editorial commentary, narrative review, or philosophical analysis rather than empirical studies.^4–8^ It has sought, among other things, to lay out a manifesto (or manifestos) for what improvement efforts might achieve, and to produce operational definitions of key terms within the field, such as those relating to quality improvement,^7^ complex interventions,^9–11^ context,^12–14^ and so on. An overlapping corpus of work is dedicated to developing the theoretical base for studies of improvement, including organisational, innovation, social and behavioural theories,^15–20^ as well as the mechanisms of change associated with quality improvement interventions.^12^ ^14^ ^21–32^ A small but important stream of work focuses on developing and testing tools to be used as part of improvement efforts, such as measurement instruments or analytical frameworks for characterisation of contexts, assessment of the impact of interventions,^33^ or determination of organisational readiness for knowledge translation.^34^

These pieces of literature make clear that the study of improvement interventions is currently an emergent field characterised by debate and diversity. One example of this is the use of the term *improvement science* which, though widely employed, is subject to multiple understandings and uses.^35^ The term is often appropriated to refer to the methods associated with Edwards Deming,^36^ including techniques, such as Plan-Do-Study-Act (PDSA) cycles and use of statistical process control (SPC) methods,^37^ ^38^ but that is not its only meaning. The *science of improvement* can also be used to refer to a broad church of research grounded in health services research, social science, evaluation studies and psychology and other disciplines. Here, Deming's methods and other established techniques for pursuing improvement may be treated as objects for inquiry, not as necessarily generating scientific knowledge in their own right.^39^ A rich social science literature is now beginning to emerge that offers important critiques of modes of improvement, including their ideological foundations^40^ ^41^ and social, ethical, professional and organisational implications,^42^ but this work is not the primary focus of this review. Instead, we offer an overview of some of the available study designs, illustrated with examples in table 1.

**Table 1:** Principles, strengths, weaknesses and opportunities for study designs for improvement interventions

Class of studies		Principles	Strengths/weaknesses	Opportunities for methodological improvement	Example
Quality improvement projects		Project is set up primarily as an improvement effort, to learn what works in a local context. It is typically motivated by a well-defined problem and oriented towards a focused aim. PDSA cycles are often applied, allowing for testing incremental, cyclically implemented changes, which are monitored through statistical process control	*Strengths:* flexibility in testing changes and adapting interventions; incorporation of knowledge generated by local improvement experiences; ability to interactively move from testing the QII locally to applying it more broadly. *Weaknesses:* generalisability of findings is not straightforward; lack of structured explanation of mechanisms of change; frequent low quality of reports	Quality improvement projects should incorporate theoretical base and qualitative methods more systematically to allow for predicting and explaining the mechanisms of change involved; more scientific vigour is needed in the application and reporting of PDSA cycles and other methods/techniques applied	An improvement initiative based on social marketing interventions developed to increase access to a psychological therapy service (especially from areas of high deprivation) involved weekly collection of geo-coded referral data and small-scale tests of change^57^ ^58^
Effectiveness studies	RCTs	RCTs may be especially suitable whenever interventions are being considered for widespread use based on their face validity and early or preliminary evidence. Differences in outcomes from delivering two or more interventions to similar groups of people or other entities are attributable to differences between the interventions. Control of confounding factors is an explicit aim	*Strengths:* direct inferences on causality. *Weaknesses:* neglect the weak boundaries separating context and intervention and the multiple interactions that take place between them; randomisation and blinding may be difficult or even not applicable; risk of contamination between groups	Improvements in the design, conducting, and reporting of RCTs are necessary to limit the high risk of bias observed currently. The awareness of the value of robust design, the need to avoid preconceived judgments about the intervention, and investments in research methods training should be pursued	A study aimed to determine the causal effects of an intervention shown effective in former pre/post studies in reducing central line-associated bloodstream infections in intensive care units.^72^
	Quasiexperimental designs	The intervention is implemented and followed-up over time, ideally with a control. Compared with a RCT, the investigator keeps more control over the intervention, but has less control over confounding factors	*Strengths:* often more practical to conduct than an RCT. *Weaknesses:* causality is not inferred directly, and confounding factors’ effects may not be obvious	Whether they have controls or not, quasiexperimental studies will be more powerful if they involve multiple measurements before and after the intervention is applied	A before-after study with concurrent controls sought to evaluate an intervention to reduce inpatient length of stay and considered the effect of the reduction on patient safety^80^
	Observational (longitudinal) studies	The implementation of the intervention is observed over time	*Strengths:* cases in practice may be the focus of the study; may be especially useful in the evaluation of sustainability of interventions. *Weaknesses:* inferences about causality may be challenging	Can be useful when other studies are not possible. They must be longitudinal and, ideally, prospective. The absence of an explicit control in the study design may be compensated by statistical techniques	A study aimed to examine the sustainability of an in-hospital quality improvement intervention in AMI, including the identification of predictors of physician adherence to AMI-recommended medication^87^
	Systematic reviews	Combining findings/samples from RCTs and quasiexperimental studies on the effectiveness of an intervention allows for more robust and generalisable QII effectiveness results	*Strengths:* ability to generate more powerful evidence. *Weaknesses:* uncritical incorporation and interpretation of studies may lead to inadequate conclusions; low use of meta-analyses	The development of systematic reviews on the effectiveness of QIIs has grown. It needs more critical appraisal of the studies included, more meta-analyses, and to deal with complex interventions in diverse contexts	Systematic review with meta-analysis aimed at assessing the effects of QIIs on the management of diabetes^88^
Process evaluations		Understanding what an intervention is in practice important, especially when the aim is to attribute effects to it	*Strengths:* process evaluations make possible an understanding of improvement interventions in practice and the fidelity with which they are implemented	Process evaluations should be embedded in effectiveness studies to capture failures in the QII implementation, and to better understand how QIIs’ components act. They need also to be more oriented towards validating theory-informed strategies	Process evaluation of a cluster randomised controlled trial aimed to examine which components of two hand hygiene improvement strategies were associated with increased nurses’ hand hygiene compliance^70^
Qualitative studies		It is not enough to know that an expected change happened or did not. It is important to understand why and how	*Strengths:* ability to capture, considering different points of view, the extent that interventions are implemented, and to explain mechanisms of change involved, based on theories	Qualitative studies should be included in quality improvement projects and QIIs’ quantitative evaluative studies for better understanding of outcomes and explanation of mechanisms of change involved.	Study that developed an ex post theory of the Michigan Intensive Care Unit project to explain how it achieved its effects^101^
Economic evaluations		It is important to know that an intervention is effective and also that the investment required is justifiable	*Strengths:* adds information about how justifiable the QII is in face of the investment required	In the literature, studies dedicated to economic evaluations of healthcare QIIs are still lacking, and there is recognition that there should be more of them in the field	Cost-effectiveness analysis of a multifaceted intervention to improve the quality of care of children in district hospitals in Kenya^104^

AMI, acute myocardial infarction; PDSA, Plan-Do-Study-Act; QII, quality improvement intervention; RCTs, randomised controlled trials.

In exploring further how improvement efforts might be studied, it is useful to distinguish, albeit heuristically, between quality improvement projects, where the primary goal is securing change, and other types of studies, where the primary goal is directed at evaluation and scientific advance (table 1). Of course, the practical and the scientific are not necessarily opposites nor in conflict with each other, and sometimes the line dividing them is blurry. Many studies will have more than one aim: quality improvement projects may seek to determine whether something ‘works’, and effectiveness studies may also be interested in producing improvement. The differences lie largely in the primary motives, aims and choice of designs.

### Quality improvement projects

A defining characteristic of quality improvement projects is that they are established primarily (though not necessarily exclusively) as improvement activities rather than research directed towards generating new knowledge: their principal aim and motive is to secure positive change in an identified service. Such projects are typically focused on a well-defined problem, are oriented towards a focused aim, and are highly practical and often, though not exclusively, local in character.

Many, though by no means all, quality improvement projects use process improvement techniques adapted from industry, such as Lean, Six Sigma and so on. Such projects are often based on incremental, cyclically implemented changes^4^ with PDSA cycles a particularly popular technique. PDSA aims to select, implement, test and adjust a candidate intervention^4^ ^43^ ^44^ to identify what works in a local context, allow interventions that do not work to be discarded, and to enable those that appear promising to be optimised and customised. The interventions themselves may be based on a range of inputs (eg, the available evidence base, clinical experience and knowledge of local context). Interventions derived from PDSA cycles can, in principle, be tested in different settings in order to produce knowledge about implementation and outcomes beyond the context of origin.^7^

In a typical quality improvement project (including those based on PDSA), measurement and monitoring of the target of change is a key activity, thus enabling quality improvement (QI) projects, if properly conducted, to be self-evaluating in some sense. SPC is often the method of choice for analysis of data in quality improvement work.^45^ SPC maps variations over time,^46^ seeking to combine ‘the power of statistical significance tests with chronological analysis of graphs of summary data as they are produced’.^47^ It is usually designed into an improvement effort prospectively, but can also be used retrospectively to evaluate time-series data for evidence of change over time.

SPC, in brief, comprises an approach to measurement in improvement initiatives as well as a set of statistical tools (control charts, run charts, frequency plots and so on) to analyse and interpret data with a view to taking action. It is especially well-suited to dealing with the dynamic, iteratively evolving nature of improvement work, in contrast with methods more oriented towards statistical hypothesis-testing relating to clearly defined and bounded interventions. It recognises that many clinical and organisational processes are characterised by some inherent random variation, and, in the context of an improvement initiative, it seeks to identify whether any observed change is due to this inherent variation (known as ‘common-cause variation’) or something different (such as the intervention, and known as ‘special-cause variation’).

Among the tools, control charts are popular for picturing the data trend and providing explicit criteria for making decisions about common-cause and special-cause variations. Different types of control charts are constructed based on different statistical distributions to account for different types of data,^48^ ^49^ but in their simplest form they plot the values of a variable of interest from measurements made regularly over time, and are typically annotated to show when various events occurred (such as the baseline period and the introduction of an intervention). They include a horizontal line showing the average of a measure over particular periods of time. Control limits, lower and upper, are set usually at ±3 SDs of the distribution the data is assumed to follow. Attention is then given to determining whether values outside the control limit indicate (with very small probability of error) that a change has occurred in the system,^47^ ^50^ ^51^ using ‘rules’ that allow detection of deviations in the measure that are unlikely to be due to normal variation. For example, baseline measurement may show that the time between prescription and dispensing medicines to take home demonstrates inherent variability that can be described as ‘common cause’; it is the normal level of variability in the process. When a rule is broken (indicating that a deviation has occurred) an investigation may reveal the underlying special cause. For example, the special cause might be the introduction of an intervention (such as staff training) that appears to be implicated in improvement or deterioration. If no rules are broken, the system is said to be in statistical control: only common-cause variation is being exhibited.

Guidance on the number of data points required is available, including the minimum number of events as a function of average process performance, as well as on the types of control charts needed to deal with infrequent events, and on the construction and interpretation of rules and rule breaks.^45^ ^49^ This is important, because care has to be taken to ensure that a sufficient number of data points are available for proper analysis, and that the correct rules are used: a control chart with 25 time points using 3SD control limits has an overall false positive probability of 6.5%.^47^ A control chart with too few data points may incur a type I error, suggesting that an intervention produced an effect on the system when it did not. Type II errors, where it is mistakenly concluded that no improvement has occurred, are also possible. Care is also needed in using SPC across multiple sites, where there may be a need for adjusting for differences among sites (requiring more formal time-series analysis), and in the selection of baseline and postintervention time periods: this should not be done arbitrarily or post hoc, as it substantially increases the risk of bias.

Attribution of any changes seen to the intervention may be further complicated by factors other than the intervention that may interfere with the system under study and disrupt the pattern of data behaviour. Qualitative or quantitative investigations may be needed to enable understanding of the system under study. Qualitative inquiry may be especially valuable in adding to the understanding of the mechanisms of change, and identifying the reasons why particular interventions did or did not work.^52^

Quality improvement projects may be published as quality improvement reports. These reports are a distinctive form of publication, taking a different form and structure from most research reports in the biomedical literature and guided by their own set of publication guidelines.^53^ QI reports provide evidence of the potential of quality improvement projects to produce valuable results in practice, particularly in local settings.^54–58^ They may be especially useful in providing ‘proof of concept’ that can then be tested in larger studies or replicated in new settings. However, quality improvement projects, and their reports, are not unproblematic. Despite their popularity, the fidelity and quality of reporting of PDSA cycles remain problematic,^59^ and the quality of measurement and interpretation of data in quality improvement projects is often strikingly poor. Further, the claims made for improvement are sometimes far stronger than is warranted:^60^ control charts and run charts are designed not to assume a sample from a fixed population, but rather a measurement of a constantly changing cause system. It is this property that makes them well suited to evaluation of improvement initiatives,^38^ but caution is needed in treating the outputs of quality improvement projects as generalisable new knowledge.^2^ ^35^ ^44^

A further limitation is that many improvement projects tend to demonstrate relatively little concern with the theoretical base for prediction and explanation of the mechanisms of change involved in the interventions. Theories of change in quality improvement reports are often represented in fairly etiolated form, for example, as logic models or driver diagrams that do not make clear the underlying mechanisms. The lack of understanding of what makes change happen is a major challenge to learning and replication.^61^

### Evaluative studies

Evaluative studies can be distinguished from quality improvement projects by their characteristic study designs and their explicit orientation towards evaluation rather than improvement alone. Some are conceived from the outset as research projects: they are motivated by and set out explicitly to test a hypothesis or otherwise generate new knowledge. Other studies are evaluations of improvement projects where the study is effectively ‘wrapped around’ the improvement project, perhaps commissioned by the funder of the improvement project and undertaken by evaluators who are external to and independent of the project.^62^ These two categories of evaluative projects are, of course, not hard and fast, but they often constrain which kind of study design can be selected. The available designs vary in terms of their goals, their claims to internal and external validity, and the ease with which they are feasible to execute given the stubborn realities of inner and outer contexts of healthcare.

*Randomised controlled trials* (RCT) randomly allocate participants to intervention and control groups, which are then treated identically apart from the intervention. Valued for their potential ability to allow for direct inferences about causality, trials in the area of improvement are typically pragmatic in character, since the interventions are generally undertaken in ‘real world’ service settings. RCTs may be especially suitable whenever interventions are being considered for widespread use based on their face validity and early or preliminary evidence.^63^ For improvement work, they are often costly and not always necessary, but they remain highly relevant to quality improvement for their ability, through randomisation, to deal with the effects on the outcomes of important unknown confounders related to patients, providers and organisations.^64^ They may be especially important when being wrong about the effectiveness of an intervention likely to be widely deployed or mandated as highly consequential, either because of the cost or the possible impact on patients.

RCTs are, of course, rarely straightforward to design and implement,^65–68^ and features of trials that may be critical in the context of medicinal products, such as randomising, and single or double-blinding, may either be impractical or irrelevant when intervening in health service delivery, while others, such as blinding of assessors, will remain essential. RCTs in health services also encounter problems with contamination within and between institutions, and with persuading sites to take part or to engage in randomisation, especially if they have strong previous beliefs about the intervention. Though some of these problems can be dealt with through study design, they remain non-trivial.

*Cluster randomised trials* have been advocated by some as an alternative to the classical RCT design for studying improvement interventions.^69–72^ These designs seek to randomise centres or units rather than individuals, thus helping to avoid some of the contamination that might occur when randomisation occurs within settings. The design does, for technical reasons, require a larger sample size.^73^ Other things being equal, a large number of small clusters is better than a small number of large clusters, but increasing the number of clusters may be very expensive. The design also makes analyses of results more complex, since the assumption of independence among observations, on which classical statistical methods rely, is not secure.^64^ ^65^ ^74^

Variants such as *stepped wedge* and others may also be used, each with strengths and disadvantages in terms of their practical operationalisation and the inferences that can be made.^64^ ^65^ ^75^ The stepped wedge trial design is especially promising as an approach to evaluating improvement interventions. A highly pragmatic design, it consists of a sequential roll-out of an intervention to clusters (organisations) so that all clusters receive the intervention by the end of the study.^76^ The stepped wedge design has many strengths, including its reassurance to organisations that none will be deprived of the intervention, reducing resistance to being randomised to a control group. It is particularly advantageous when logistical, practical, or financial constraints mean that implementing the intervention in a phased way will be helpful, and it can even be used as part of a pragmatic, non-funded approach to intervention implementation. On the more negative side, it is likely to lead to a longer duration of trial period than more conventional designs, and additional statistical complexity.^75^

Despite the promise of trial designs for evaluating quality improvement interventions, the quality of studies using these methods has often been disappointing. A relatively recent systematic review of 142 trials of quality improvement strategies or financial incentives to improve the management of adult outpatients with diabetes, identified that nearly half the trials were judged to have high risk of bias, and it emphasised the need to improve reporting of quality improvement trials.^77^ One major challenge to the deployment of trials in the study of improvement is that improvement interventions may tend to mutate over time in response to learning, but much trial methodology is based on the assumption of a stable, well-defined intervention, and may not give sufficient recognition to the interchange between intervention and context.

*Quasi-experimental* designs^64^ ^65^ may be an attractive option when trials are not feasible, though they do mean that investigators have less control over confounding factors. Quasiexperimental designs often found in studies of improvement^64^ ^65^ include uncontrolled and controlled before-and-after studies, and time-series designs.

*Uncontrolled before-and-after studies* are simple. They involve the measurement of the variables of interest before and after the intervention in the same-study sites, on the assumption that any difference in measurement ‘after’ compared with ‘before’ is due to the intervention.^64^ ^65^ Their drawback is that they do not account for secular trends that might be occurring at the same time,^66^ something that remains an important problem determining whether a particular intervention or programme has genuinely produced improvement over change that was occurring anyway.^78^ ^79^

*Controlled before-and-after studies* offer important advantages over uncontrolled ones. Their many strengths in the study of improvement^66^ ^80^ include an increased ability to detect the effects of an intervention, and to control for confounders and secular trends, particularly when combined with difference-in-difference analyses.^62^ ^81^ However, finding suitable controls is often not straightforward.^64–66^ ^80^ ^82^ A frequent problem resulting in inadequate controls is selection solely on the basis of the most superficial structural characteristics of healthcare units, such as size, teaching status, location, etc. The choice of relevant characteristics should also be made based on the anticipated hypotheses concerning the mechanisms of change involved in the intervention, and the contextual influences on how they work (eg, informatics, organisational culture, and so on). Looking at the baseline quality across organisations is also fundamental, since non-comparable baselines or exposure to secular trends may result in invalid attribution of effects to the intervention(s) under evaluation.

*Quasi-experimental time-series designs and observational longitudinal designs* rely on multiple successive measurements with the aim of separating the effect of the intervention from secular trends.^83^ ^84^ One question that often arises is whether and when it might be more advantageous to time-series analysis instead of the SPC methods characteristic of QI projects that we discussed earlier. SPC techniques can indeed monitor trends, but are challenging in studies involving multiple sites given the difficulty of adjusting for confounding variables among sites. A QI project in a small microsystem (eg, a hospital ward) usually has small sample sizes, which are offset by taking many measurements. A large-scale effort, such as a QI collaborative deploying a major QI intervention might, however, be better off leveraging its larger sample sizes and using conventional time-series techniques. Other statistical techniques for longitudinal analysis may also allow for identifying changes in the trends attributable to the intervention, accounting for the autocorrelation among observations and concurrent factors.^64–66^ ^85^ ^86^ Observational longitudinal designs may be especially useful in the study of sustainability of quality improvement.^87^

*Systematic reviews* of improvement studies, whether or not they include meta-analyses, are now beginning to appear,^88–92^ and are likely to play an important role in providing overviews of the evidence supporting particular interventions or methods of achieving change. Such reviews will require considerable sophistication; low quality and contradictory systematic reviews may result without thoughtful, non-mechanical appraisal of the studies incorporated, detailed descriptions of the interventions and implementation contexts, and consideration of combinations of multiple components and their interactions. Use of methods for synthesis that allow more critique and conceptual development may be especially useful at this stage in the emergence of the field.^93^ ^94^

The study of improvement interventions should not, of course, be limited to quantitative assessments of the effectiveness of interventions. The field of programme evaluation is a rich but underused source of study designs and insights for the study of improvement interventions. Dating back to the 1960s, this field has identified both the benefits and the challenges of deploying traditional, epidemiologically derived experimental methods in the evaluation of social interventions.^95^ ^96^ It developed mainly in the context of evaluating social programmes (including those in the area of welfare, justice and education), and it tends to be pragmatic about what is feasible when the priority is programme delivery rather than answering a research question, about the influence of external contexts, and about the mutability of interventions over time.

Programs are nowhere near as neat and accommodating as the evaluator expects. Nor are outside circumstances as passive and unimportant as he might like. Whole platoons of unexpected problems spring up.^97^

The programme evaluation field has urged a theory-driven approach to evaluation, one that, as well as determining whether something works, also seeks to explicate the underlying mechanisms, or how it works.^98^ It thus offers many lessons for those conducting studies of improvement initiatives and projects, including the need to attend to what happens when a programme or intervention is implemented (known as process evaluation), and the fidelity with which it was implemented. Carol Weiss's list of the basic tasks of evaluation^99^ (box 2), for example, remains highly salient for those studying improvement work in healthcare.

Box 2 **Carol Weiss's logic of analysis in evaluation^99^**

- **What went on in the programme over time? *Describing*.**
  A. Actors
  B. Activities and services
  C. Conditions of operation
  D. Participants’ interpretation
- **How closely did the programme follow its original plan? *Comparing*.**
- **Did recipients improve? *Comparing*.**
  A. Differences from preprogramme to postprogramme
  B. (If data were collected at several time periods) Rate of change.
  C. What did the improvement (or lack of improvement) mean to the recipients?
- **Did recipients do better than non-recipients? *Comparing*.**
  A. Checking original conditions for comparability
  B. Differences in the two groups preprogramme to postprogramme
  C. Differences in rates of change
- **Is observed change due to the programme? *Ruling out rival explanations*.**
- **What was the worth of the relative improvement of recipients? *Cost-benefit or cost-effectiveness analysis*.**
- **What characteristics are associated with success? *Disaggregating*.**
  A. Characteristics of recipients associated with success
  B. Types of services associated with success
  C. Surrounding conditions associated with success
- **What combinations of actors, services and conditions are associated with success and failure? *Profiling*.**
- **Through what processes did change take place over time? *Modelling*.**
  A. Comparing events to assumptions of programme theory
  B. Modifying programme theory to take account of findings
- **What unexpected events and outcomes were observed? *Locating unanticipated effects*.**
- **What are the limits to the findings? To what populations, places and conditions do conclusions not necessarily apply? *Examining deviant cases*.**
- **What are the implications of these findings? What do they mean in practical terms? *Interpreting*.**
- **What recommendations do the findings imply for modifications in programme and policy? *Fashioning recommendations*.**
- **What new policies and programmatic efforts to solve social problems do the findings support? *Policy analysis*.**

*Process evaluations* are an especially important feature of the evaluation of improvement interventions. Such evaluations make possible the exploration of the components of interventions and the fidelity and uniformity of implementation, as well as testing hypotheses concerning mechanisms of change associated with intervention components, refining theory and improving strategy effectiveness.^70^ Ideally, they should be embedded in studies of effectiveness, adding information to clarify whether the target population actually received the planned activities, experiences of those charged with delivering the intervention as well as those receiving it, and what factors inhibited or promoted effectiveness.^70^ Process evaluations can combine a range of study methods and cross-sectional or longitudinal designs, including surveys among managers, frontline healthcare professionals and patients, and the measurement of variables, through interviews, direct observation or medical record review.

Use of *qualitative methods* is invaluable in enabling the understanding of what form a quality improvement intervention takes in practice, as well as providing data about why and how the planned activities succeed or not.^100^ Using methods such as interviews, ethnographic observation, and documentary analysis, qualitative studies may be able to capture the extent that the interventions are implemented with fidelity at different organisational levels, and to explicate the mechanisms of change involved. The ‘triangulation’ of data collection and interpretation using quantitative and qualitative approaches makes the findings more reliable and powerful.^62^ An explicit grounding in formal theory is likely to support fuller understanding of how the interventions are expected to make a difference, and to contribute to building a knowledge base for improvement. Social science theory combined with the use of qualitative methods is particularly useful for bringing to the surface implicit theories of change held by practitioners, and for distinguishing empirical facts from normative judgements.^101^

Finally, *economic evaluations* of quality improvement interventions, such as those focused on clinical interventions or healthcare programmes, are mainly concerned with appraising whether the differential investment in an intervention is justifiable in face of the differential benefit it produces.^102–106^ Quality improvement investments compete with other possible applications of healthcare resources, and economic analyses are necessary to inform rational decisions about interventions to invest in to produce the greatest benefits, and even whether the resources would be better allocated to other social purposes. Contrary to commonly held assumptions, quality improvement efforts, especially those focused on safety, may not be cost-saving, possibly because of the fixed costs of a typical healthcare setting; QI may generate additional capacity rather than savings.^107^ Studies are, however, still lacking with, for example, few good-quality comparative economic analyses of safety improvement strategies in the acute care setting, possibly, in part, because of the additional methodological challenges associated with their evaluation.^108^ ^109^ ^110^

## Conclusions

This review has identified a wide range of study designs for studying improvement in healthcare. Small-scale quality improvement projects remain a dominant approach, but need to be conducted and reported better, and appropriate caution exercised in treating the data from such projects as equivalent to research-standard evidence. The epidemiological paradigm offers a range of experimental, quasi-experimental, and observational study designs that can help in determining effectiveness of improvement interventions. Studies using these designs typically seek to determine whether an improvement has occurred, and if so, whether it can be attributed to the intervention(s) under study; these methods are less well suited to investigating questions of ‘why’ or ‘how’ any change occurred. They are most powerful when they allow for measurements over time and control for confounding variables. But such studies, particularly those using more experimental designs, are often difficult to conduct in the context of many improvement activities. Interventions that are purposefully evolving over time, as is a common feature of quality improvement interventions, lack many of the stable characteristics generally assumed for studies of effectiveness. Trial-based designs may under-recognise the weak boundaries separating context and intervention, and the multiple interactions that take place between them. Given the complex role played by context in quality improvement, external validity may be very difficult to establish. Quantitative and qualitative methodological approaches can play complementary roles in assessing what works, how, and in what contexts,^111^ and the field of programme evaluation has remained under-exploited as a source of methods for studying improvement. Programme evaluation is especially important in stressing the need for theoretically sound studies, and for attention to implementation and fidelity of interventions.

Much could be achieved by improving the rigour with which existing designs are applied in practice, as can be seen from the example of PDSA cycles. Too often, PDSA cycles are contrived as a form of pilot testing rather than formal steps guided by explicit *a priori* theories about interventions, too often they are reported as a ‘black box’, too often measurement strategies are poor and do not comply with even basic standards of data collection and interpretation, and too often reported claims about the magnitude of improvement are not supported by the design. These limitations act as threats both to internal and external validity, and risk the reputation of the field as well as thwarting learning. At the very least, great care needs to be taken in making claims about the generalisability or achievements of such projects.

As the study of improvement develops, reconciling pragmatism and scientific research rigour is an important goal, but trade-offs need to be made wisely, taking into account the objectives involved and the inferences to be made. There is still much to explore, and quantitative and qualitative researchers will have important and complementary roles in dealing with many yet-unanswered questions.^90^ ^100^ ^111–114^
